# Integrating Single-Cell and Bulk RNA Sequencing Reveals the Malignant Phenotype of CBX4 in Prostate Cancer

**DOI:** 10.7150/jca.115613

**Published:** 2025-07-28

**Authors:** Zihao Liu, Yang Liu, Zhinan Fu, Hua Huang, Runpeng Wang, Zhun Wang, Shuanghe Peng, Jiahao Wang, Ziqi Fang, Liwei Liu, Ruibing Chen, Yong Wang

**Affiliations:** 1 Department of Urology, The Second Hospital of Tianjin Medical University, Tianjin, 300211, China.; 2 Tianjin Institute of Urology, the Second Hospital of Tianjin Medical University, Tianjin, 300211, China.; 3 School of Pharmaceutical Science and Technology, Tianjin University, Tianjin, 300072, China.; 4 Department of pathology, The second hospital of Tianjin medical university, Tianjin, 300211, China.

**Keywords:** CBX4, prostate cancer, single-cell sequencing, PI3K/AKT signaling

## Abstract

**Background:** The expression pattern and functions of CBX4 in prostate cancers remain ambiguous. This study aims to investigate the performance of CBX4 in prostate cancer progression and preliminary inquiry potential mechanisms.

**Methods:** The GEPIA data website was utilized to evaluate the expression patterns of CBX families and their correlations with prognosis. The “clusterprofiler” package was used for GSEA analysis. Seurat and CellChat package were used to analyze the single-cell expression profiles. The RT-qPCR, western blot and IHC staining were performed to detect the expression of CBX4 in prostate cancer tissues or cell lines. The cell functional experiments were performed, including MTT, colony formation assay, Transwell assay and scratch assay. Western blot was conducted to explore the regulation of CBX4 on EMT markers and PI3K/AKT pathway markers.

**Results:** CBX4 was significantly up-regulated at tissue and cell levels in prostate cancer. High expression level of CBX4 was closely associated with advanced stage and poor prognosis. Of note, CBX4 was observed to promote immunosuppressive tumor environment via PDGF, VEGF, WNT signaling by cell-cell communications. *In vitro* experiments confirmed the expression level. Cell function and western blot proved the down-regulation of CBX4 dramatically inhibited the proliferation, invasion and migration of prostate cancer cells by targeting PI3K/AKT signaling.

**Conclusion:** CBX4 might serve as a potential oncogene in prostate cancer progression. This study provides a new target for the treatment of prostate cancer.

## Introduction

Prostate cancer (PCa) is considered as one of the most common malignances for male around the world and also remains to be a threaten to cancer death 1, 2. In recent studies, many genetic and epigenetic changes have been revealed that contribute to prostate cancer progression 3. Yao et al. demonstrated that metabolic reprogramming mediated by Lon protease 1 (*LONP1*) could promotes the progression of prostate cancer 4. Besides, a latest study indicated that the long noncoding RNA LncZBTB10 was able to promote prostate cancer progression and abiraterone resistance via regulation of androgen receptor (*AR*) 5. However, genetic research into prostate cancer is still limited and more research is needed.

Polycomb group (PcG) proteins play a critical role in gene regulation by enabling the stable inheritance of cellular states 6, 7. Dysregulated epigenetic control mediated by PcG proteins has been extensively linked to various cancers 8, 9. As core components of PcG, Chromobox (*CBX*) family proteins are pivotal in cancer progression and tumorigenesis by maintaining tumor suppressor functions and preserving undifferentiated cancer stem cell states 10. The human CBX family comprises eight members (*CBX1-8*), each featuring conserved N-terminal chromodomains 11. Structurally, CBXs are divided into two subgroups: the heterochromatin protein 1 (*HP1*) subfamily (*CBX1/3/5*) and the polycomb subfamily (*CBX2/4/6/7/8*) 12. The CBX family members are essential elements of epigenetic regulation and perform significant roles in the development and advancement of multiple cancers 13. For example, CBX6 and CBX7 have been identified as prognostic biomarkers of bladder cancer 14. Chen et al. reported that* CBX8* promoted the proliferation and metastasis of lung cancer by regulating *CDKN2C* and *SCEL* 15. In prostate cancer, the dysregulation of CBX families were also demonstrated that *CBX2*, *CBX3*, *CBX4*, and *CBX8* were upregulated, while *CBX6* and *CBX7* were downregulated 13. However, the underlying mechanisms and deeper functional roles remain to be fully elucidated.

## Materials and methods

### Cell culture and clinical specimens

This study utilized common prostate cancer cell lines (LNCaP, PC-3, C4-2 and DU-145), one normal prostate cell line (RWPE-1), all of which were sourced from the Cell Bank of the Chinese Academy of Sciences (Shanghai, China). Cells were maintained at 37°C in a 5% CO_2_ atmosphere using corresponding mediums.

Prostate cancer tissue samples were provided by Second Hospital of Tianjin Medical University (SHOTMU). The study included 12 pairs of prostate cancer tissues and their matched normal tissues. All the included cases were confirmed not to have other malignant tumors. The study protocol was approved by the Institutional Review Board of SHOTMU, and written informed consent was obtained from all participants.

### Transcriptome analyses

The expression profiles and clinical information of prostate cancer were downloaded by 'TCGAbiolinks' (v2.26.0) package. The gene differential expression analysis was performed by 'limma' (v3.54.2) package, and the genes with adjusted P < 0.05 were thought as differential expressed genes. The Kaplan-Meier curves analyzed by survminer (v0.4.9) package were used to evaluated the prognostic value of CBX family. UALCAN database was used to investigate the correlations between clinical characteristics and the expression level of *CBX4* 16. We divided the expression level of CBX4 into high and low CBX4 groups according to the median value, and completed the difference analysis of the two groups using limma (v3.54.2) package. The fold change of each gene was used as the input file for GSEA analysis. Gene set enrichment analyses were performed by 'clusterProfiler' (v4.6.2) package.

### Single-cell RNA sequencing analysis

A total of 8 single-cell RNA sequencing samples obtained from GSE193337 (4 normal and 4 prostate cancer samples) was included in the study. The conventional single-cell process was adopted, including removal of double cells, standardization, removal of batch effect, dimensionality reduction clustering. The details of data processing could be referred to our previous published research 17. The Cell marker database (http://117.50.127.228/CellMarker/) was used to annotate each cell clusters. Nonlinear dimensionality reductions were utilized to visualize the distance among cell clusters. The cell-cell communications were analyzed by 'Cellchat' (v1.6.1) package.

### Reverse transcription-quantitative polymerase chain reaction (RT-qPCR)

Total RNA was extracted from tissues or cells using TRIzol Reagent (Life Technologies), following the manufacturer's standard protocols. mRNA expression levels were quantified using the GoTaq qPCR Master Mix (Promega, Madison, WI, USA). Relative gene expression fold changes were calculated using the 2^ΔΔCt^ method. All primer sequences used in this study are listed below:

CBX4 forward primer: GCAGAGTGGAGTATCTGGTGA; CBX4 reverse primer: AGCTTGGCACGGTTGTCAG.

GAPDH forward primer: GGAGCGAGATCCCTCCAAAAT; GAPDH reverse primer: GGCTGTTGTCATACTTCTCATGG.

### MTT assay

The transfected cells were plated in 96-well plates at a density of 2 × 10³ cells per well and cultured for 48 hours. Following incubation, 10 μL of MTT reagent was added to each well, and the plates were incubated for an additional 4 hours. The medium was then removed, and the resulting formazan crystals were solubilized using 150 μL of DMSO. Absorbance was measured at 570 nm using a microplate reader (Bio-Rad, Richmond, CA, USA) to determine cell viability at specified time points.

### Colony-formation assays

For the colony-formation assay, 500 cells were plated in each well of a 6-well plate and cultured for three weeks at 37°C. After incubation, colonies were fixed with 4% paraformaldehyde (PFA) for 30 minutes at room temperature and stained with 2% crystal violet solution for 15 minutes. The wells were subsequently washed with PBS and air-dried. Colonies were quantified manually under a microscope.

### Migration and invasion assays

Cell invasion was assessed using Matrigel-coated Transwell chambers (BD Biosciences, USA). Briefly, 6 × 10⁴ cells were suspended in serum-free medium and seeded into the upper chambers, while the lower chambers were filled with medium containing 20% FBS as a chemoattractant. After 14-36 hours of incubation, cells that migrated through the membrane were fixed and stained using a three-step staining kit (Thermo Scientific, USA). The stained cells were imaged and quantified under a light microscope (Olympus, Japan) at 100× magnification.

For the cell scratch assay, transfected cells were cultured in 6-well plates for 48 hours to achieve confluency. A sterile 10 μL pipette tip was used to create a uniform scratch in each well. Cells were then maintained in serum-free medium to eliminate the influence of FBS on migration. Scratch images were captured at 0 and 24 hours using a light microscope (Olympus, Japan). The migration distance was measured as the ratio to the initial scratch width (0 hours) and analyzed.

### Western blot analysis

Cells were lysed using RIPA buffer supplemented with 1 mM PMSF (Solarbio, Beijing, China). Protein concentrations were quantified using the BCA Protein Assay Kit (Thermo Fisher Scientific). Protein samples were denatured by boiling, resolved on 10% SDS-PAGE gels, and subsequently transferred onto polyvinylidene fluoride (PVDF) membranes (Millipore). The membranes were blocked with 5% (w/v) skimmed milk for 1 hour at room temperature, followed by overnight incubation with primary antibodies at 4°C. After three washes with TBST (Tris Buffered Saline with Tween), the membranes were incubated with horseradish peroxidase (HRP)-conjugated secondary antibodies for 1 hour at room temperature. Protein bands were visualized using enhanced chemiluminescence (ECL) reagent (Millipore). The antibodies involved was listed as follow: CBX4 (Immunoway, YT7583), GAPDH (Proteintech, 60004-1-Ig), E-cadherin (Cell Signaling Technology, 3195), N-cadherin (Cell Signaling Technology, 13116), Vimentin (Cell Signaling Technology, 5741), Snail (Cell Signaling Technology, 3879), PI3K (Abclona, A22730), AKT (Abclona, A17909), mTOR (Proteintech, 66888-1-Ig), c-myc (Immunoway, YM8143).

### Immunohistochemistry (IHC)

Prostate cancer tissues or normal tissues were fixed in formalin and paraffin-embedded. Consecutive sections (4 μm thick) were prepared using a rotatory microtome. Following deparaffinization, hydration, antigen retrieval, and blocking of endogenous peroxidase activity, the slides were incubated with specific primary antibodies overnight at 4°C. Subsequently, the slides were treated with HRP-conjugated secondary antibodies for 1 hour at room temperature. Color development was achieved using diaminobenzidine (DAB), and the slides were counterstained with hematoxylin. Stained sections were examined under an inverted microscope imaging system (Olympus).

The expression levels of CBX4 were independently assessed by two pathologists in a blinded manner. Staining intensity was graded on a scale of 0 (no staining), 1 (weak), 2 (moderate), or 3 (strong). The percentage of positively stained tumor cells was categorized as 0 (< 10%), 1 (10-25%), 2 (26-50%), 3 (51-75%), or 4 (> 75%). The final immunostaining score, ranging from 0 to 12, was calculated by multiplying the intensity score by the percentage score. A total score of 1-3 was defined as low expression, and ≥ 4 was defined as high expression.

### Statistical Analysis

Data analysis was performed using SPSS 24.0 (IBM, Armonk, NY, USA). Continuous variables are presented as mean ± standard deviation (SD). Group comparisons were conducted using either one-way ANOVA or Student's t-test, as appropriate. A p-value of less than 0.05 was considered statistically significant.

## Results

### The expression pattern of CBX family in prostate cancer

**Figure 1:**
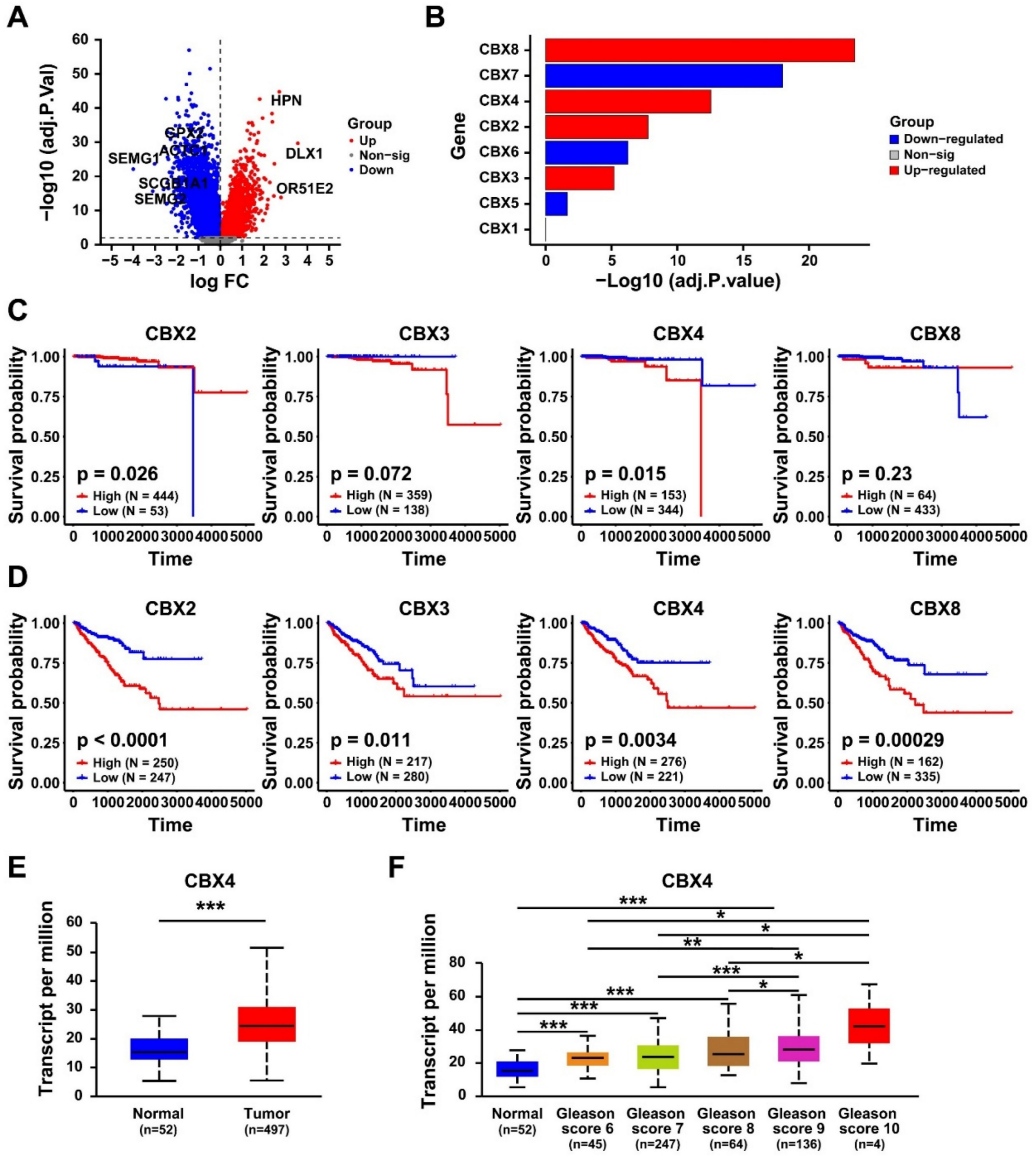
** The expression pattern of CBX family in prostate cancer. A.** Volcano map of gene expression in prostate cancer. **B.** Expression of CBX family (1-8) in prostate cancer. **C.** The Overall survival analysis of CBX2, CBX3, CBX4 and CBX8 in prostate cancer patients. **D.** The Progression Free Interval analysis of CBX2, CBX3, CBX4 and CBX8 in prostate cancer patients. **E.** The expression of CBX4 in prostate tumor tissues and normal tissues analyzed by GEPIA website. **F.** Gleason Score analysis of CBX4 in prostate cancer.

To investigate the differential genes between normal prostate and tumor tissues, the differential gene analysis was performed. As shown in Figure 1A, a total of 10683 significant genes was identified. Then we further clarified the expression of CBX family members in prostate cancer, which result showed that *CBX5*, *CBX6* and *CBX7* were significantly down-regulated in prostate cancer tissues, while *CBX3, CBX2, CBX4* and *CBX8* was notably up-regulated (Figure 1B). To evaluate the correlation between up-regulated genes and prognosis of prostate cancer patients, the survival analysis was performed. The Overall survival (OS) analysis showed that only higher expression of *CBX4* resulted in a worse OS (Figure 1C). However, the result of Progression Free Interval (PFI) analysis showed that all these genes exhibited significant correlations with PFI (Figure 1D). Synthesize the above analysis results, *CBX4* was considered as a potential oncogene, and selected for the following analysis. The GEPIA analysis showed that compared to normal tissues, *CBX4* was indeed up-regulated in prostate cancer tissues (Figure 1E). More detailly, the higher expression level of *CBX4* was also positively associated with higher Gleason Score of prostate cancer (Figure 1F). These findings indicate that *CBX4* was high-expressed in prostate cancer and correlated with prognosis.

### CBX4 was associated with cancer-related pathways

**Figure 2:**
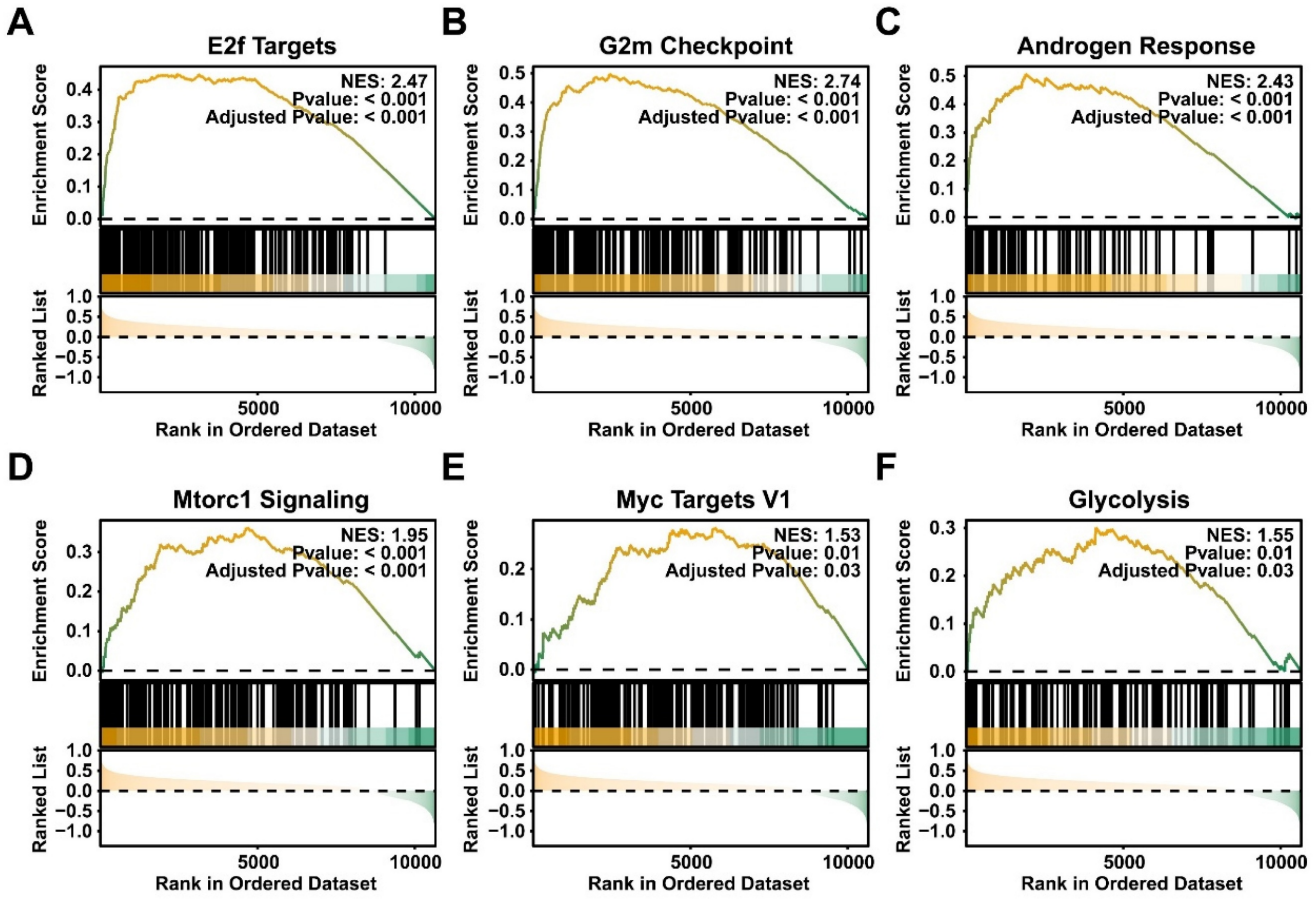
** CBX4 was associated with cancer-related pathways.** The related pathways of CBX4 analyzed by the Gene Set Enrichment Analysis, including **A.** E2F targets, **B.** G2M check point, **C.** Androgen response, **D.** mTORC1 signaling,** E.** Myc targets V1, **F.** Glycolysis. NES, Normalized enrichment score.

The differential expressed genes between high- and low- CBX4 patients were identified (Table S1). The Gene Set Enrichment Analysis was performed to identify the related pathways and biological functions of CBX4. It should be noted that NES > 0 indicates a significant upregulation of the pathway in the CBX4 high-expression group. The result showed that CBX4 was positively correlated with E2F targets (Figure 2A), G2M check point (Figure 2B), and Androgen response (Figure 2C), mTORC1 signaling (Figure 2D), Myc targets V1 (Figure 2E) and Glycolysis (Figure 2F). These related pathways played essential roles in cancer progression, thus *CBX4* might also serve as a critical character in tumor development.

### Validation of CBX4 expression in single-cell RNA level

**Figure 3:**
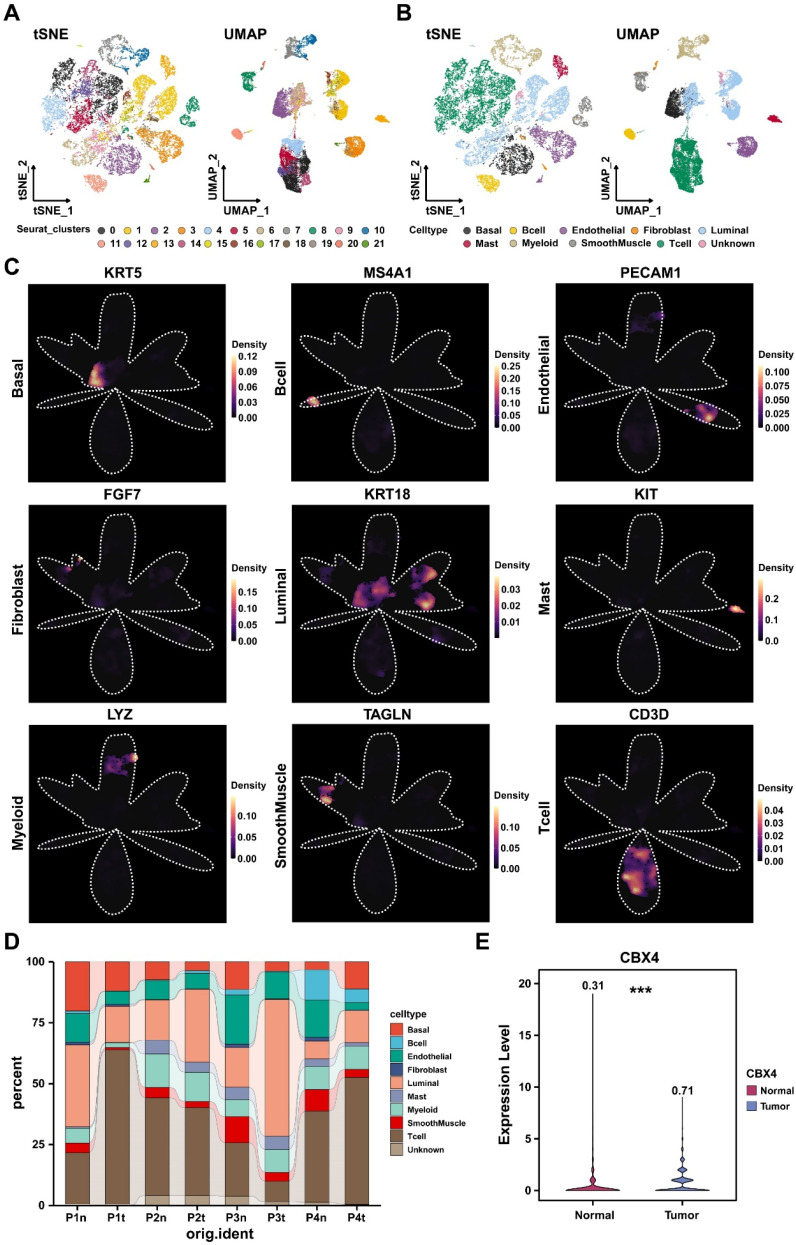
** CBX4 was upregulated at single cell level. A-B.** The cell clusters and cell types in normal prostate and prostate cancer tissues demonstrated using T- distributed stochastic neighbor embedding (t-SNE, **A**) and uniform manifold approximation and projection (UMAP,** B**); **C.** The density of cells with specific marker were illustrated using UMAP. The color represents the density level of the cells with the marker; **D.** The proportion of different cell types in prostate cancer samples. **E.** The boxplot showing the expression level of CBX4 of luminal cells in normal and tumor tissues.

The above results suggested the expression level and involved potential downstream pathways of *CBX4*. Next, we further explored the expression level of *CBX4* at single-cell level. As shown in Figure 3A, a total of were included and clustered into 22 distinct cell clusters. After careful annotations by CellMarker database, nine main cell type including Basal cells, B cells, endothelial cells, fibroblasts, luminal cells, mast cells, myeloid cells, smooth muscle cells, and T cells were identified (Figure 3B). The density plots exhibited the markers used for cell annotations (Figure 3C). The cell components of each sample were illustrated in Figure 3D. luminal cells have been shown to be the source of prostate cancer cells 18. Therefore, we analyzed the expression level of *CBX4* in luminal cells. As shown in Figure 3E, the expression level of luminal cells in tumor tissues was significantly higher than that in normal prostate tissues. In summary, *CBX4* was upregulated in prostate cancer at both tissues and cell levels.

### CBX4 played an immunosuppressive role in tumor microenvironment

**Figure 4:**
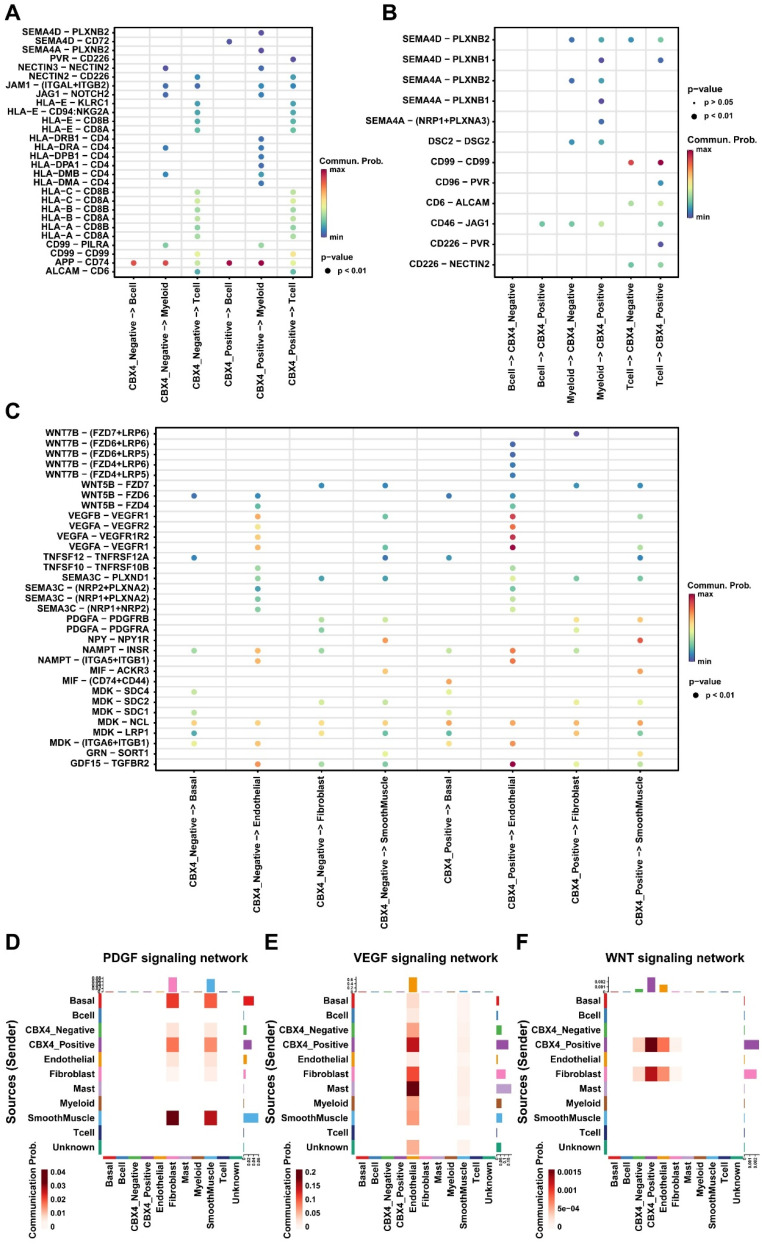
** CBX4 was associated with immunosuppressive tumor microenvironment. A.** Dot plot showing cell-cell contact signaling from CBX4 positive and CBX4 negative cells to immune cells; **B.** Dot plot showing cell-cell contact signaling from immune cells to CBX4 positive and CBX4 negative cells. **C.** Dot plot showing secreting signaling from CBX4 positive and CBX4 negative cells to immune cells; **D-F.** Heatmaps showing the signaling network of PDGF (**D**), VEGF (**E**), and WNT (**F**) among all cell types.

To further explore the role of CBX4 in the microenvironment of prostate cancer. Luminal cells were first divided into negative and positive groups based on *CBX4* expression levels, and then cell communication analysis was performed. We first focused on the interaction between luminal cells and immune cells. As shown in Figure 4A, we found that* CBX4*-positive cells sent stronger signals to myeloid cells than negative cells, and these signals mainly included *HLA-CD4* receptor ligand pairs. However, there was no significant difference in the interaction between B cells and T cells and luminal cells. Interestingly, myeloid cells, as ligands, also showed significantly enhanced SEME4A signal communication with *CBX4*-positive cells, while interaction with *CBX4*-negative cells was less intense (Figure 4B). Subsequently, the interactions of luminal cells with other cells were also analyzed. Interestingly, we observed that WNT and VEGF signaling from *CBX4*-positive cells was stronger than that from *CBX4*-negative cells, especially for endothelial cells. This suggests that *CBX4* may be associated with angiogenesis and immunosuppression in the tumor microenvironment. In addition, the communication of PDGF, VEGF, and WNT among all cell components is shown in Figure 4D-F. The results showed that *CBX4*-positive cells had more extensive and stronger interactions with stromal cells, including endothelial cells, smooth muscle cells, and fibroblasts, than *CBX4*-negative cells. Taken together, these findings reveal the potential tumor-promoting role of *CBX4* in prostate cancer.

### CBX4 was up-regulated in prostate cancer tissues and cell lines

**Figure 5:**
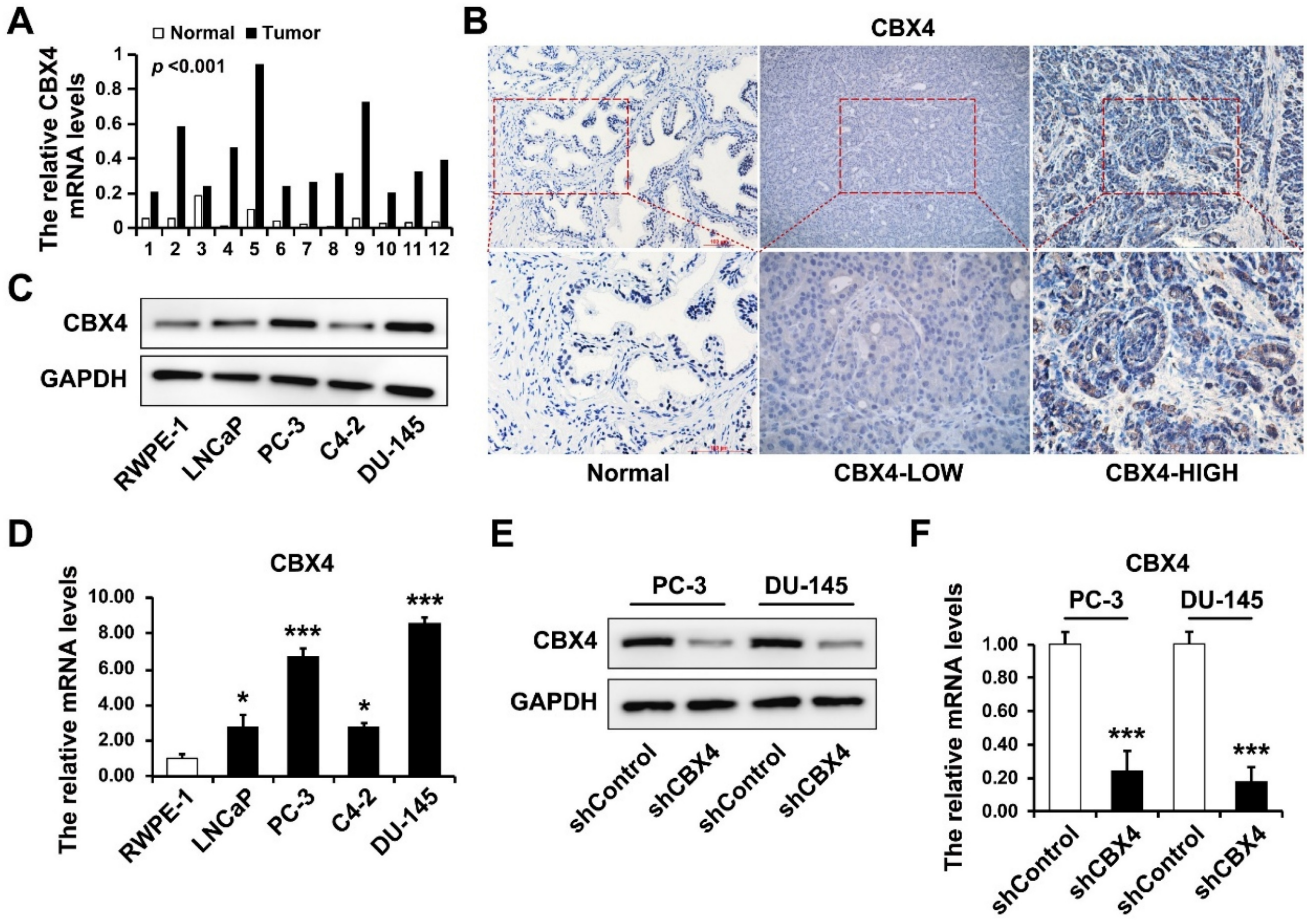
** CBX4 was up-regulated in prostate cancer tissues and cell lines. A.** The mRNA levels of CBX4 in 12 paired prostate cancer tissues and normal tissues detected by RT-qPCR.** B.** The expression of CBX4 detected by IHC.** C.** The protein expression levels of CBX4 in prostate cancer cells detected by Western blot. **D.** The mRNA levels of CBX4 in prostate cancer cells detected by RT-qPCR. The construction of CBX4-downregulated cell lines and verified by **E.** Western blot and **F.** RT-qPCR.

The *CBX4* mRNA was extracted from 12 paired prostate cancer tissues and normal tissues, and detected by RT-qPCR, which result showed that *CBX4* mRNA was dramatically up-regulated in tumor tissues (Figure 5A). Then the IHC experiment was performed to further visualize the expression of *CBX4* in tissues, indicating that the expression of *CBX4* is heterogeneous in prostate cancer tissues, however *CBX4* had almost no staining in normal tissues (Figure 2B). Besides, the protein expression of CBX4 was determined by western blot in normal prostate cell RWPE-1 and prostate cancer cells, including LNCap, PC-3, C4-2 and DU-145 cells, which showed that compared to RWPE-1, CBX4 was up-regulated in prostate cancer cells, especially in PC-3 and DU-145 cells (Figure 5C). At the meantime, the RT-qPCR analysis obtained the same results (Figure 5D). Thus, PC-3 and DU-145 cell lines were selected for CBX4 knock down. The shCBX4 plasmid was infected into these two cells to construct CBX4 down-expressed stable cell lines and verified by western blot (Figure 5E) and RT-qPCR (Figure 5F).

### Down-regulation of CBX4 inhibited prostate cancer progression

**Figure 6:**
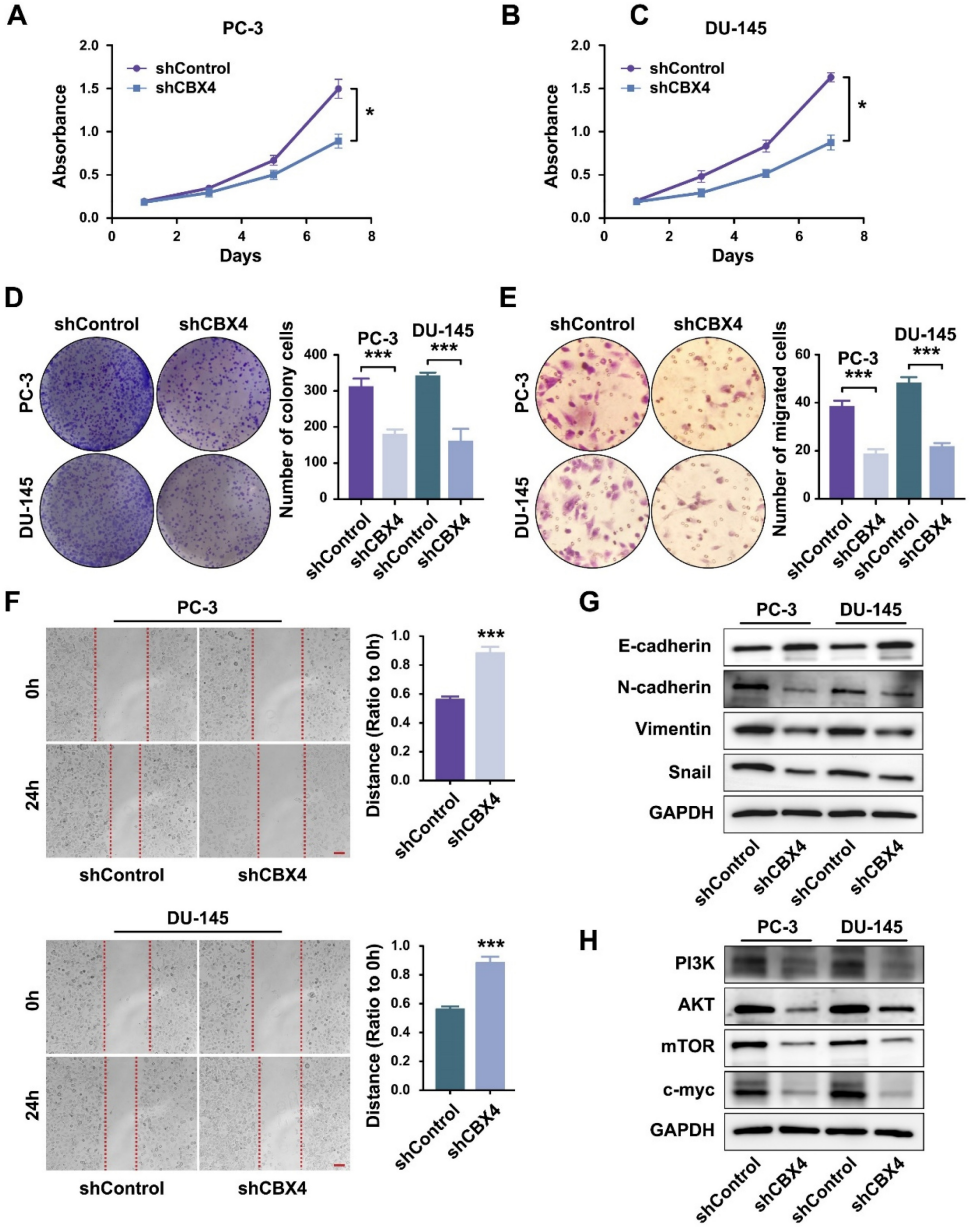
** Down-regulation of CBX4 inhibited prostate cancer progression. A.** The proliferation rate of shCBX4 and shControl in PC-3 cells.** B.** The proliferation rate of shCBX4 and shControl in DU-145 cells. **C.** The colony formation assay performed in PC-3 and DU-145 cells.** D.** The transwell assay performed in PC-3 and DU-145 cells. **E.** The scratch assay performed in PC-3 and DU-145 cells. **F.** The expression levels of EMT-related markers, including N-cadherin, E-cadherin, Vimentin and Snail, detected by Western blot. **G.** The expression levels of PI3K-AKT signaling related markers, including PI3K, AKT, mTOR and c-myc, detected by Western blot.

Then the cell functional experiments were performed to identify the role of CBX4 in prostate cancer development. The MTT results showed that the proliferation rates of cells were significantly inhibited in *CBX4* down-regulated PC-3 cells (Figure 6A) and DU-145 cells (Figure 6B). Besides, the colony formation assay indicated that down regulating CBX4 resulted in fewer colony numbers and smaller sizes (Figure 6C). Then, the invasion abilities of prostate cancer cells were detected by Transwell assay, which result showed that the migrated cells decreased after *CBX4* knocking down (Figure 6D). The scratch assay further proved that knocking down *CBX4* could inhibit the migration of prostate cancer cells (Figure 6E). As for the dramatic performance of *CBX4* on cell invasion and migration, we hypothesized that CBX4 might play a role in markers related to epithelial-mesenchymal transition (EMT). The western blot assay showed that down-regulation of* CBX4* led to decreased expressions of N-cadherin, Vimentin and Snail, but an increase in expression of E-cadherin (Figure 6F). Finally, according to the previous conclusions of GSEA enrichment analysis, we preliminarily verified the regulatory relationship between *CBX4* and PI3K signaling pathway, which result showed that shCBX4 inhibited the expression levels of markers in PI3K/AKT signaling, including PI3K, AKT, mTOR and c-MYC (Figure 6G). These findings indicated that CBX4 served as a potential oncogene in prostate cancer, the down-regulation of* CBX4* significantly inhibited the proliferation, invasion and migration of prostate cancer cells, which might act through the PI3K/AKT signaling pathway.

## Discussion

The dysregulation of gene families has been reported in cancer process 19-21. As a critical member of the CBX protein family, *CBX4* has emerged as a multifaceted regulator in tumorigenesis and cancer progression. In hepatocellular carcinoma (HCC), CBX4 has been reported to induce nuclear translocation of YAP1 protein and regulated tumorigenicity and stem-like properties of HCC cells 22. Besides, in colorectal cancer, CBX4 exhibits tumor-suppressive effects by recruiting histone deacetylase 3 (*HDAC3*) to the* Runx2* promoter 23. In prostate cancer, Zheng et al. demonstrated that lncRNA *RAMS11* promoted cell growth and metastasis by *CBX4* complex via binding to Top2α 24. However, more detailed studies on the role and mechanism of *CBX4* in prostate cancer are still lacking. In this study, we identified *CBX4* from the expression pattern analysis of CBX family members in prostate cancer and verified its association with prognosis of prostate cancer patients, indicating that *CBX4* served as a tumor promoter. These findings indicated that CBX4 could be a potential biomarker for prostate cancer, and it is necessary to further explore its role in tumor microenvironment.

Recent advances in single-cell RNA sequencing (scRNA-seq) have transformed our understanding of tumor biology by enabling unprecedented resolution in dissecting intratumoral heterogeneity, tumor microenvironment interactions, and dynamic cellular states during cancer progression 25-27. Emerging innovations like spatial transcriptomics are further expanding their potential to revolutionize cancer diagnosis and therapy selection 28, marking a new era in precision oncology where cellular-level insights directly inform patient management 29. In this study, we analyzed 24259 cells to further investigate the expression level of *CBX4* at single-cell level, which results showed that the expression level of luminal cells in tumor tissues was significantly higher than that in normal prostate tissues. This finding further validated our statement at the single-cell level.

The tumor immune microenvironment (TIME) plays a pivotal role in prostate cancer progression, therapeutic resistance, and immune evasion 30. So far, accumulating studies have shown that *CBX4* has emerged as a regulator of TIME in cancers. Wang et al. reported that *CBX4* expression was induced in tumor-infiltrating CD8^+^T cells and inhibited CD8^+^T cell function by regulating glucose metabolism in tumor tissue 31. Besides, Matthew et al. found that *CBX4* is a critical regulator of the cancer stem cell (CSC) phenotype in squamous cell carcinomas of the skin and hypopharynx, which closely related to the major component of the microenvironment 32. However, the relationship between *CBX4* and the tumor microenvironment in prostate cancer has not been reported. In this study, we focused on the interaction between luminal cells and immune cells, as well as other cells, which showed that *CBX4*-positive cells had more extensive and stronger interactions with stromal cells including fibroblasts, endothelial cells and smooth muscle cells, indicating *CBX4* may promote the suppressive tumor microenvironment. Importantly, we observed the PDGF, VEGF, and WNT signaling showing the high communication probability between stromal cells and *CBX4*-positive cells, which has never reported. These results reveal the potential molecular mechanism of *CBX4* in the tumor microenvironment, providing a theoretical basis for the treatment of prostate cancer targeting *CBX4*.

In summary, our research confirmed that CBX4 is significantly highly expressed in both prostate cancer tissues and cells and is associated with a poor prognosis of prostate cancer. To our knowledge, this study reports for the first time that CBX4 may promote the formation of the tumor microenvironment through the PDGF, VEGF and WNT signaling pathways in the tumor microenvironment. Finally, *in vitro* experiments preliminally confirmed that the tumor-promoting effect of CBX4 was related to PI3K and AKT signaling pathways.

Obviously, our study also had some limitations. For instance, the sample size we collected for bioinformatics analysis is limited, more external datasets should be included in the subsequent study. Further research is still needed to delineate the specific role and mechanism of CBX4 in prostate cancer microenvironment.

## Conclusion

In this study,* CBX4* was found to be up-regulated in prostate cancer and related to worse prognosis. Down regulation of* CBX4* could inhibit the malignant phenotypes of prostate cancer cells and inhibited the markers of EMT and PI3K-AKT signaling.

## Supplementary Material

Supplementary table.
